# Understanding health-related quality of life of informal carers in amyotrophic lateral sclerosis: a scoping review and conceptual framework

**DOI:** 10.1186/s12955-025-02427-2

**Published:** 2025-09-29

**Authors:** Rosie Bamber, Jill Carlton, Christopher McDermott, Theocharis Stavroulakis

**Affiliations:** 1 Division of Neuroscience, Sheffield Institute for Translational Neuroscience (SITraN), School of Medicine and Population Health, University of Sheffield, 385a Glossop Rd, Sheffield, S10 2HQ UK; 2 Sheffield Centre for Health and Related Research (SCHARR), School of Medicine and Population Health, University of Sheffield, Regent Court, 30 Regent Street, Sheffield, S1 4DA UK

**Keywords:** Amyotrophic lateral sclerosis, Motor Neuron Disease, Caregiving, Caring, Carer, Quality of Life, Health-Related Quality of Life, Conceptual Framework, Person Reported Outcome Measure

## Abstract

**Background:**

Amyotrophic Lateral Sclerosis (ALS) is a rapidly progressive, life-limiting neurodegenerative disease. Informal carers provide extensive support, significantly impacting their health-related quality of life (HRQoL). Current HRQoL measurement using person-reported outcome measures (PROMs) in ALS carers lacks consistency and comprehensiveness, hindering robust assessment and synthesis. There is evident need for a comprehensive conceptual framework of HRQoL, to fully capture the multidimensional nature of caregiving in ALS. Such a framework is essential to inform research and clinical practice, ensuring relevant measurement and meaningful clinical discussions. This study aimed to develop this evidence-based framework.

**Methods:**

This study comprised two stages. Firstly, a scoping review was undertaken in March 2024 using Medline, Embase, and CINAHL to identify primary articles exploring HRQoL in ALS carers. Qualitative, mixed methods and quantitative articles using multi-item PROMs to assess HRQoL in informal ALS carers were included. Relevant themes and subthemes were extracted from articles and PROMs and mapped onto an existing conceptual framework for people with ALS (Quality of Life in ALS, QuALS), which covers physical, psychological, and social HRQoL domains in people with ALS. The Carer-QuALS framework was subsequently developed and refined using existing literature and consultation with ALS carers. PROMs within this review were then indexed against the finalised Carer-QuALS framework.

**Results:**

From 715 search results, 82 articles and 44 PROMs were eligible for inclusion. One new subtheme ‘physical caring activities’ emerged, while seven subthemes lacked support from the literature. In three structured consultation sessions, nine ALS carers, reviewed the draft Carer-QuALS framework (consisting of seven themes and 43 subthemes). Based on their input, one new subtheme ‘privacy’ was added, six subthemes were removed, and one was retained, despite lacking support from review literature. The final Carer-QuALS framework includes 37 subthemes: 8 physical, 6 social, and 23 psychological.

**Conclusions:**

This review presents a comprehensive conceptual framework encompassing the multidimensional impact of ALS caregiving on the HRQoL of informal carers. The framework provides a resource that can be used by researchers, clinicians, and patient advocacy groups for multiple purposes (e.g., to support PROM selection to measure HRQoL, to guide future PROM development, and to facilitate discussions between informal carers and clinicians).

**Supplementary Information:**

The online version contains supplementary material available at 10.1186/s12955-025-02427-2.

## Background

Amyotrophic Lateral Sclerosis (ALS) is an incurable, adult-onset neurodegenerative condition characterised by progressive weakness of limb, bulbar and respiratory muscles [1]. Progressive muscle weakness leads to rapid functional decline and reduced ability to perform daily tasks independently [2]. Death, typically due to respiratory failure, commonly occurs within two to five years from onset [3]. In the absence of a cure, treatment primarily focuses on symptom management and preservation of quality of life [3] (QoL), given the profound impact of ALS on both function and QoL for those living with this condition [4].

Support with basic care needs in ALS [5] is frequently provided at home by a family member [6]with no prior caregiving experience [7]. These ‘informal’ carers, provide unpaid care support, whether physical and/or non-physical, over any caregiving duration [8]. In addition to physical care, carers often manage the cognitive and behavioural changes that affect 50% of people with ALS at diagnosis [9]. These non-motor symptoms deteriorate over the disease course [10] and can significantly increase carer strain over and above physical symptoms [11] and may lead to increased carer burden [11–13]. Caregiving in ALS is recognised to negatively affect carers’ own health and QoL [14]. While there is growing evidence supporting the need to integrate support for carers within healthcare service delivery [8, 15]this remains an ongoing challenge for healthcare professionals [16].

Health-related quality of life (HRQoL) is a subjective multi-dimensional concept that encompasses the effect of health state on physical, psychological and social functioning [17, 18]. Given its subjectivity, different methodological approaches to understanding and measuring HRQoL are evidenced within existing literature, including, qualitative [19]quantitative [20] and mixed-methods [21] designs. Qualitative approaches focus on understanding the lived experiences of carers in ALS, whilst quantitative approaches typically employ Person-Reported Outcome Measures (PROMs) to quantify HRQoL. These measures provide a subjective assessment, rated via an individual or proxy (e.g., clinician). PROMs may explore one aspect of functioning, or a specific symptom within HRQoL, for example depression (i.e., a component of psychological function). Alternatively, PROMs may cover all aspects of functioning within HRQoL (i.e., physical, psychological and social aspects). Existing use of PROMs to measure informal carer HRQoL in ALS varies widely [22]underscoring the lack of consensus about best practices in both research and clinical practice. Such inconsistency may compromise ability to synthesise findings, monitor change over time, and implement supportive interventions.

There is a notable gap in the availability of HRQoL PROMs specifically designed for carers in ALS, and existing tools have been shown to lack comprehensiveness for this cohort [22]. Therefore, use of existing HRQoL PROMs risk underestimating the multidimensional and unique impact of caregiving in ALS on their own HRQoL. Additionally, terminal diseases are known to underlie distinct caregiver experiences [2]and in rare diseases, such as ALS, condition-specific HRQoL PROMs can demonstrate increased sensitivity [23]. To address this, there is a need for a comprehensive conceptual framework of HRQoL for ALS carers, grounded in the lived experiences and priorities of carers. Such a framework could inform the selection of appropriate HRQoL PROMs for ALS carers, guide development of future PROMs, and facilitate meaningful discussion between carers and healthcare professionals. Conceptual frameworks serve to define core constructs under investigation and map relationships within concepts [24]using written descriptions and/or visual representations [25]. By capturing the multidimensional nature of caregiving in ALS, a comprehensive framework will enhance understanding of its impact on carers’ HRQoL and improve the relevance and utility of HRQoL PROMs used in this context.

The aim of this study was to develop a comprehensive, evidence-based conceptual framework (herein referred to as Carer-Quality of Life in ALS [Carer-QuALS]) for HRQoL for carers in ALS and to map the content of existing PROMs used with ALS carers to this framework. This study had five objectives: (1) to conduct a scoping review to identify articles that have assessed ALS carer HRQoL (or an aspect thereof) either quantitatively (using PROMs) or qualitatively, (2) to map the content of PROMs and qualitative articles to an a priori HRQoL framework, (3) to develop a draft conceptual framework of HRQoL for ALS carers, (4) to refine the draft framework following feedback from ALS carers, and (5) to index the content of identified PROMs and qualitative articles to the Carer-QuALS framework to provide a resource for researchers and clinicians to identify potential instruments for measuring HRQoL for ALS carers and guide future PROM development.

## Methods

**Fig. 1:**
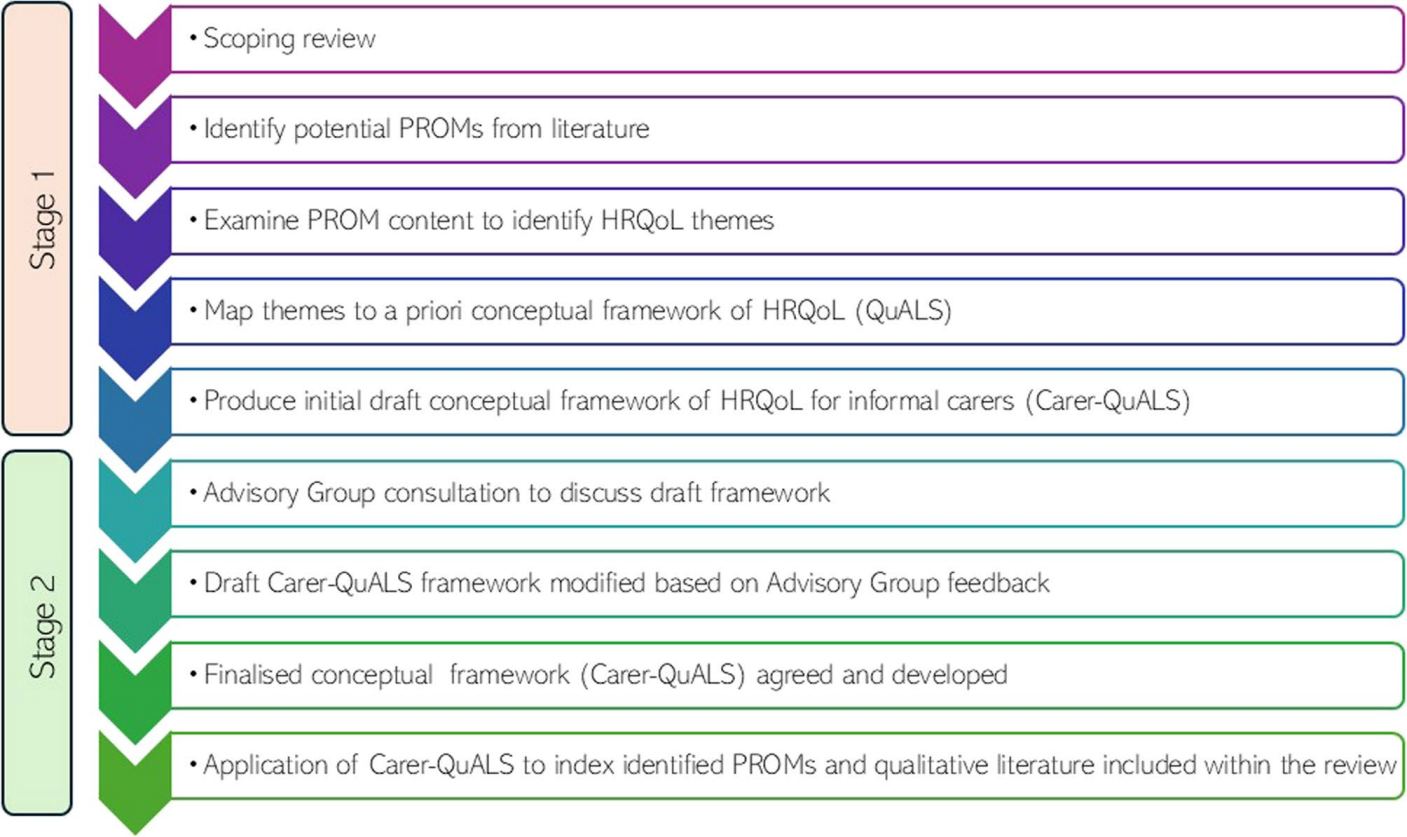
Carer-QuALS development. Pictorial representation of multi-stage methods employed in the development of the comprehensive Carer-QuALS conceptual framework. Carer-QuALS = Carer Quality of Life in Amyotrophic Lateral Sclerosis

This study involved two stages. In Stage 1, a scoping review was undertaken to explore existing literature for qualitative, quantitative or mixed-methods articles that have investigated HRQoL (or an aspect thereof) in carers in ALS. The aim of the review was to identify HRQoL themes and subthemes (i.e., concepts relating to physical, psychological or social functioning) from qualitative data and content of PROMs used to measure HRQoL in carers. In Stage 2, a conceptual framework (Carer-QuALS) was developed. Themes identified in Stage 1 were considered against a priori conceptual framework, that reflects HRQoL for individuals with ALS. The draft conceptual framework was discussed with an ALS carer Advisory Group and refined before finalisation. Detailed methodology is outlined below and represented in Fig. 1.

### Stage 1: scoping review

#### Search strategy

The scoping review was conducted according to guidance from the Joanna Briggs Institute [26] and reported according to the PRISMA guidance for scoping reviews (PRISMA-ScR) [27]. A protocol was developed and registered on ORDA, the University of Sheffield research data repository [28]. An information specialist aided the development of a comprehensive search strategy. One researcher conducted searches of MEDLINE (via Ovid), EMBASE (via Ovid) and CINAHL (via EBSCO) on 8th March 2024. Databases were searched from inception, with no language restriction. Syntax was tailored per database with a combination of keywords related to HRQoL, ALS and informal carers. Full search strategy is available in Additional File 1.

#### Article screening

Study records were extracted from databases and imported into EndNote 21 (Clarivate Analytics) for deduplication [29] and exported to Excel for screening. A hierarchical screening tool (Additional File 1) was developed according to eligibility criteria (Table 1) to support a reproducible screening strategy [30]. All records were assessed for eligibility by one researcher. A second researcher independently assessed a random sample of records (20% of titles and abstracts, and 10% of full-text articles). A conservative inclusion approach was adopted. If either researcher indicated inclusion at title or abstract level, the record was retained for consideration for full-text screening. Discrepancies regarding inclusion at full-text screening were resolved by discussion.

**Table 1:** Article inclusion and exclusion criteria

Inclusion	Exclusion
• Subjects: Adult informal carers (≥ 18) of individuals with ALS. No restrictions to race, ethnicity, geography, or socioeconomic status. • Intervention/Exposure: Assessment via a multi-item, freely available, self-report PROM measuring HRQoL or a domain of HRQoL. • Outcome: HRQoL measurement. • Articles: Primary research (qualitative, quantitative or mixed-methods design), published as a full-text original article in English. *Quantitative or Mixed-Methods Articles: Uses a freely available, multi-item self-report HRQoL PROM with adult informal carers of people with ALS. *Qualitative Articles: Mixed-population studies (e.g., including both patients and carers) if data reported separately for ALS patients and carers.	• Articles: without available full text (e.g., published abstracts). Articles including informal carers of mixed syndromic groups, unless the carer population includes more than 75% of informal carers of people with ALS, or separate data is available for informal carers of people with ALS.

### Data extraction

An article data extraction form was designed and independently piloted by two researchers. The data were extracted by one researcher and a second researcher extracted data from a random sample of 10% the included articles for comparison. An assessment of quality, critical appraisal or risk of bias was not completed as this is typically not part of scoping review methodology [26].

### PROM screening

All articles were examined to identify which PROMs had been used, and qualitative articles that did not use PROMs were retained for qualitative data extraction. Copies of all identified PROMs were sourced by the research team. The content of each PROM was independently assessed against predetermined criteria. To be included in the review, the PROMs had to be freely available, multi-item self-report PROMs or PROM subscales, available in English and measuring at least one aspect of HRQoL of carers in ALS. PROM eligibility was independently assessed by two blinded researchers; one researcher assessed all PROMs, while a second researcher assessed a random sample of 20% PROMs. Any disagreement was resolved by discussion.

### PROM characteristics

PROM characteristics were extracted by one researcher from PROMs retained in the review and gathered in a separate PROM data extraction spreadsheet (Additional File 3). This included PROM name, abbreviation, version number (if applicable), recall period, type of response option (i.e., frequency, severity, agreement), number of response options, number of subscales (if applicable), number of items, whether the PROM is preference-based (meaning utility values could be calculated for the purpose of economic evaluation), whether the PROM was developed specifically for carers, and the number of included articles the PROM was utilised within.

### Identification of HRQoL themes

To identify HRQoL themes (i.e., concepts relating to physical, psychological or social functioning), qualitative data in the form of in-text quotations, and the content of PROM items were reviewed. Themes pertinent to the HRQoL of carers in ALS were extracted and used to iteratively develop the draft Carer-QuALS framework. This began with extracting small extracts of text, or ‘codes’ from PROM items or quotes from qualitative papers. Codes were then reviewed and either categorised within existing themes from the a priori QuALS framework or grouped into new themes if not sufficiently covered within the a priori framework. One researcher reviewed all qualitative papers and PROM content, whilst a second researcher reviewed a random sample of 10% of qualitative papers and 20% of PROMs. Where disagreement regarding data extraction or theme development occurred, consensus was reached through discussion.

### Stage 2: developing the Carer-QuALS framework

The themes identified in Stage 1 were mapped against the a priori QuALS framework [31]. The QuALS framework is a comprehensive model of HRQoL for people living with ALS, developed using a similar methodological approach. It is the only conceptual framework tailored specifically to the lived experience of people living with ALS and therefore was deemed to have greater relevance to ALS carers than generic HRQoL frameworks. New themes were added to the QuALS framework to produce a draft version of the Carer-QuALS framework. Existing themes (from the QuALS framework) that were not identified in Stage 1 were highlighted for discussion with the Advisory Group. Descriptions for subthemes within the Carer-QuALS framework were drafted based on qualitative data and the content of PROM items.

### Advisory group

The draft Carer-QuALS framework was shared and discussed with members of an Advisory Group established for the project. The Advisory Group comprised nine individuals with experience of providing informal care to people living with ALS. Three online sessions, 90-minutes each in duration, were held in February 2025, with different members of the Advisory Group present at each session. The Advisory Group were given the opportunity to identify whether themes, subthemes and their descriptions were relevant, comprehensive and understandable. Within each session, the discussion of themes occurred in a different order. The Advisory Group were able to offer feedback and suggestions for amendments to inform the development of the finalised framework. Once all feedback was received within online sessions, the draft Carer-QuALS framework (and accompanying descriptions) was amended and recirculated to the Advisory Group for further consideration and feedback via email. The framework was finalised following this. Full details of the Advisory Group consultation are available in Additional File 2.

Lastly, PROMs and qualitative articles identified in this review were indexed to the finalised Carer-QuALS framework. This process entailed indexing the content of PROMs and qualitative articles to the subthemes within the Carer-QuALS framework. This was undertaken independently by three members of the research team, with any discrepancies resolved through discussion.

## Results

### Stage 1: scoping review

#### Article selection

**Fig. 2:**
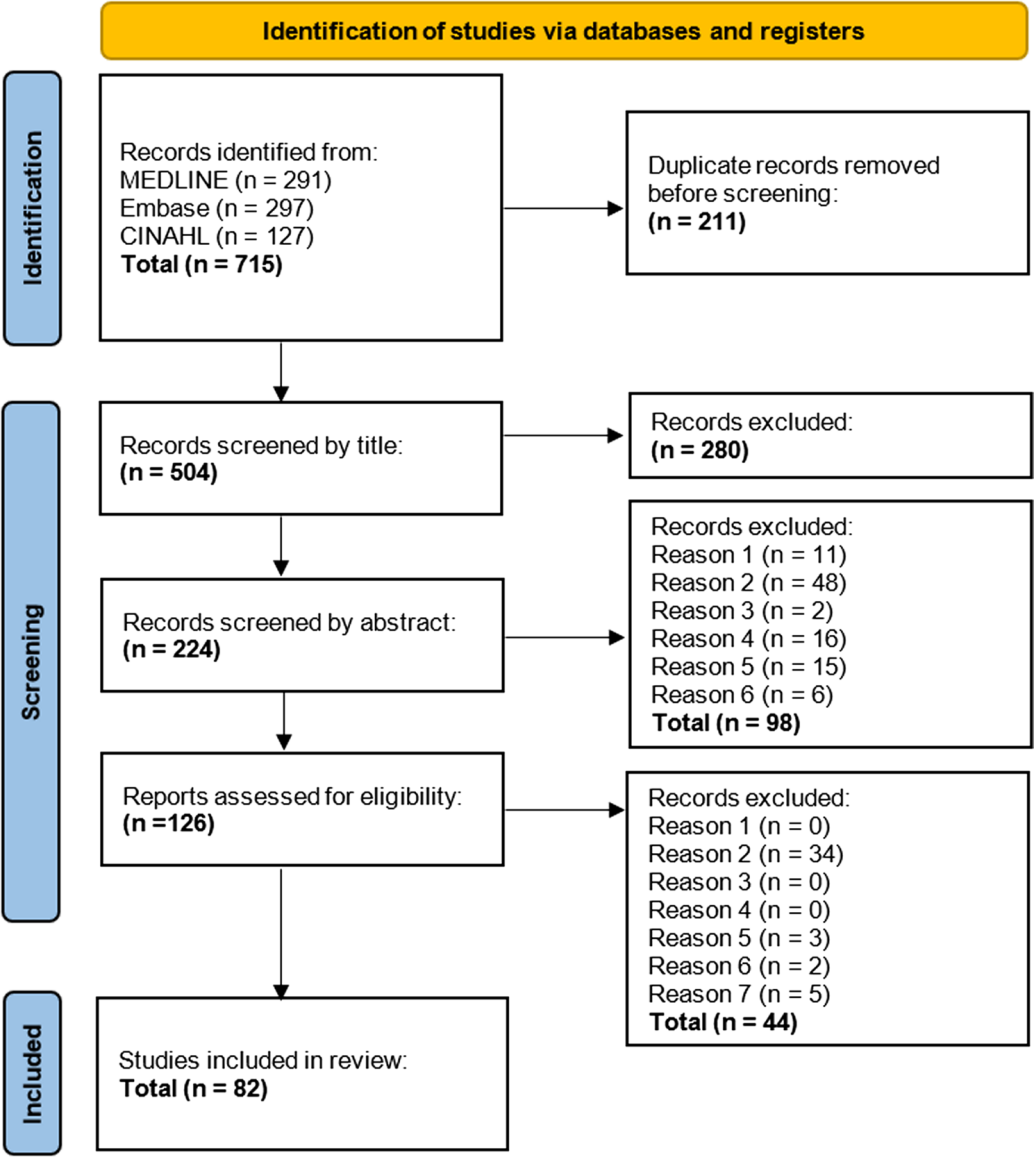
PRISMA-ScR diagram. Flowchart adapted according to PRISMA-ScR template [27]. Reasons for full text exclusions: (1) Title and abstract not written in English in a peer-reviewed journal. (2) Not a primary research paper with full-text available. (3) Study participants are not adults carers ≥ 18. (4) Study participants are not carers for individuals with ALS. (5) HRQoL, or domain/s of HRQoL are not investigated. (6) Articles with mixed syndromic care recipients have < 75% ALS patients, or separate data is not available for ALS carers. (7) Later excluded (12/09/2024) as PROM within paper excluded. CINAHL = Cumulative Index of Nursing and Allied Health Literature. MEDLINE = Medical Literature Analysis and Retrieval System Online. Instrument. PRISMA-ScR = Preferred Reporting Items for Systematic Reviews and Meta-Analyses for Scoping Reviews

The searches generated 715 records (Fig. 2). Overall, 82 articles were included in this review (67 utilising PROMs). Cohen’s kappa of inter-rater reliability was k = 0.54 for title level screening (‘moderate agreement’), k = 0.73 for abstract level screening (‘substantial agreement’) and k = 0.82 for full-texts (‘near perfect agreement’ [32]). The 82 articles, published between 2000 and 2024 across 18 countries, were predominantly observational 72/82 (87.8%), with 10/82 (12.2%) employing interventional designs. Of these, 57/82 (69.5%) were cross-sectional and 25/82 (30.5%) longitudinal. The number of informal ALS carers across all articles ranged from 8 to 434 (median = 60.5, IQR = 67.8). The gender of carers was reported in 65/82 (79.3%) articles. Of these, a mean of 68.1% were female. The gender of individuals with ALS was reported in 57/82 (69.5%) articles. Of these, a mean of 40.6% were female. The cognitive or behavioural status of the person with ALS was not reported in 47/82 (57.3%) articles. Of the 35 articles that did report this, 17/35 (48.6%) excluded individuals with cognitive or behavioural changes—and, by extension, their carers. Additional File 3 includes full details of included articles and PROMs.

### PROM selection

From eligible articles, 76 individual HRQoL PROMs were identified in quantitative and mixed-methods articles. After screening, 44 PROMs were deemed eligible for inclusion (Additional File 4). The total number of PROMs used with ALS carers per article ranged from 1 to 6 with mean of 2.2 (SD = 1.2). The most frequently used individual PROM across the quantitative and mixed-methods articles was the Zarit Burden Interview (ZBI) [33]used in 30/69 articles (43.5%), followed by the Hospital Anxiety and Depression Scale (HADS) [34]used in 22/69 articles (31.9%). In contrast, 25/44 PROMs (56.8%) were used in only a single article. Only nine PROMs identified in this review (9/44, 20.5%) were developed specifically for use in general carer populations. The Caregiver Network Scale (CNS) [35] was the only PROM identified in this review that was developed specifically for carers in ALS. A detailed breakdown of PROM usage across studies is provided in Additional File 3.

### Stage 2: developing the Carer-QuALS conceptual framework

The draft Carer-QuALS framework consisted of seven themes and 43 subthemes. A new subtheme ‘*Physical caring activities*’, that did not exist in the a priori QuALS framework, was added, and seven existing subthemes were redundant (i.e., not supported by scoping review findings). The draft framework was considered by the Advisory Group, who suggested further refinements. One new subtheme ‘*Privacy*’ was added, and six subthemes from the QuALS framework, which were not supported by scoping review findings, were removed. One existing QuALS subtheme ‘*Body image*’, was retained following Advisory Group feedback, despite being absent from scoping review findings. Advisory Group feedback was used to refine the descriptions of themes and subthemes within the finalised Carer-QuALS framework. Full details of Advisory Group feedback and amendments are reported in Additional File 5.

**Fig. 3:**
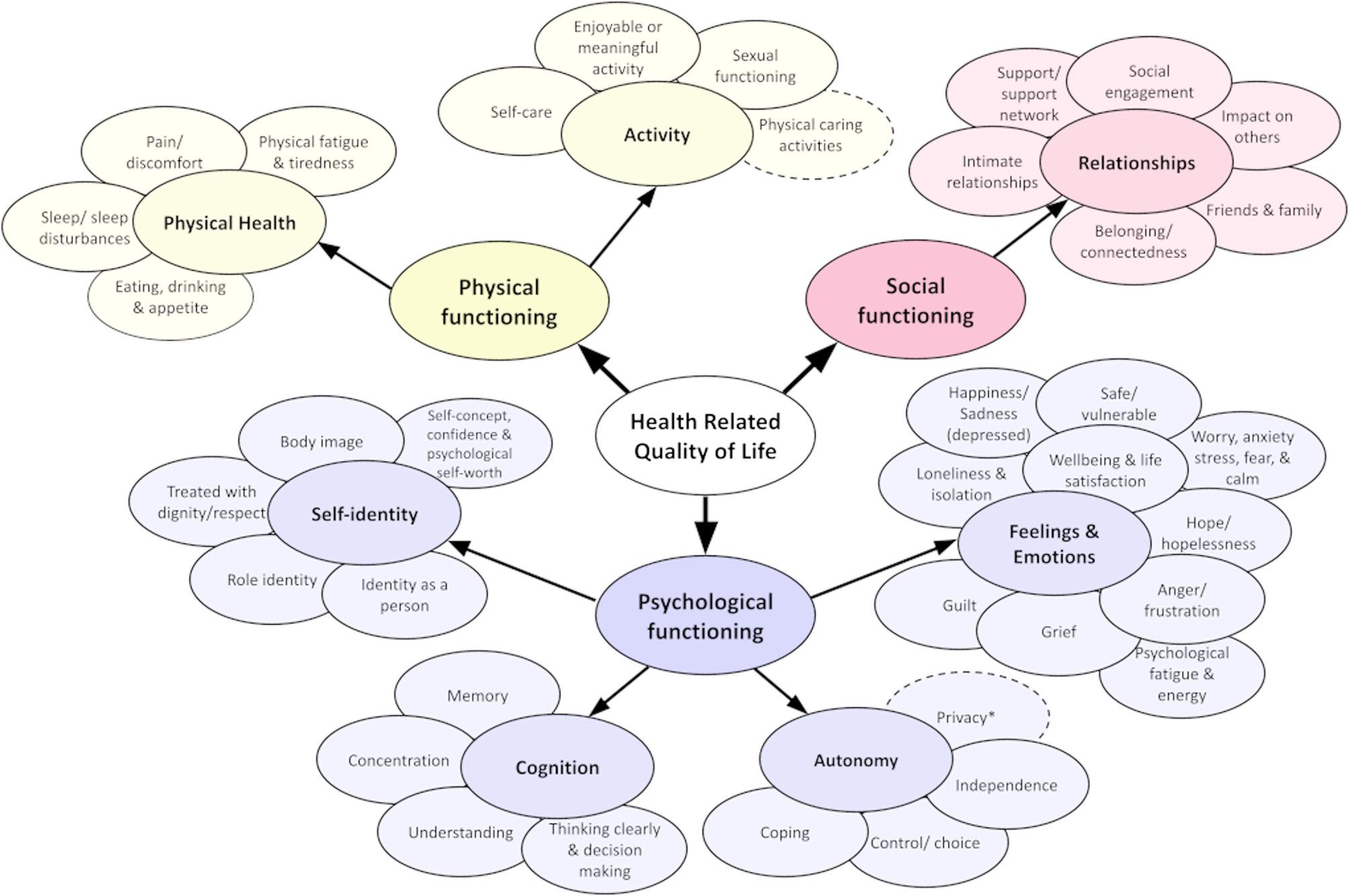
Carer-QuALS conceptual framework. Pictorial representation of the multidimensional Carer-QuALS framework, composed of physical, psychological and social domains of functioning. Seven subthemes and 37 subthemes are illustrated in coloured ovals. Ovals with a solid border were extracted from the pre-existing QuALS framework [31]. The themes and subthemes within Carer-QuALS have undergone amendments to reflect the caregiver experience in ALS. Ovals with a dotted border are new concepts added to the framework following data extraction or Advisory Group feedback with ALS carers. Subthemes with an asterisk are concepts added as a result of Advisory Group feedback alone

The finalised Carer-QuALS framework represents the multidimensional impact of caring in ALS on carers’ own HRQoL (Fig. 3). The framework has conceptual overlaps with the a priori QuALS framework, however, distinct differences were identified and developed to capture the carer perspective in ALS. The framework is structured hierarchically, beginning with the three basic domains of HRQoL: physical, psychological and social functioning, and contains seven themes with 37 subthemes; ‘*Activity*’ (*n* = 4 subthemes), ‘*Physical Health*’ (*n* = 4), ‘*Relationships*’ (*n* = 6), ‘*Self-identify*’ (*n* = 5), ‘*Cognition*’ (*n* = 4), ‘*Autonomy*’ (*n* = 4) and ‘*Feelings & Emotions*’ (*n* = 10). Descriptions of subthemes are available in Additional File 6. Key findings from the scoping review and Advisory Group feedback are described in-text below.

### Physical functioning: activity

The theme ‘*Activity*’ is divided into four subthemes: *Self-care*,* Enjoyable or meaningful activity*,* Sexual functioning*, and *Physical caring activities*. The ‘*Self-care*’ subtheme was expanded from the QuALS framework to include the concept of ‘*self-neglect*’ to reflect the experience of deprioritising one’s needs to fulfil caregiving responsibilities. The ‘*Enjoyable and meaningful activity*’ subtheme was broadened to encompass the ‘*ability to take breaks from caregiving*’, ‘*seek respite*’, and ‘*make time for oneself amidst caring duties*’. ‘*Physical caring activities*’ is a newly introduced subtheme and captures the commonly physically demanding nature of caregiving in ALS. This includes activities such as ‘*supporting transfers and mobility*’, ‘*toileting*’ and using medical devices including ventilatory aids and gastrostomy devices.

### Physical functioning: physical health

The theme of ‘*Physical Health*’ comprises four subthemes: *Pain/discomfort*,* Sleep/sleep disturbances*,* Eating*,* drinking & appetite*, and *Physical fatigue & tiredness*. The ‘*Pain/discomfort*’ subtheme was revised from the QuALS framework to remove pain concepts less relevant to carers such as cramping and joint stiffness. In the ‘*Eating*,* drinking & appetite*’ subtheme, the term ‘*swallowing*’ was removed from the title, in addition to descriptors related to choking, chewing and controlling food in the mouth. Instead, aspects of eating with others and comfort eating were added, reflecting the social and psychological dimensions of eating when caring for someone with ALS.

### Psychological functioning: autonomy

The theme of ‘*Autonomy*’ includes four subthemes: *Coping*,* Control/choice*,* Independence*, and *Privacy*. The ‘*Coping*’ subtheme includes concepts such as resilience, adapting to caring responsibilities and denial. The concepts of ‘*avoidance*’, ‘*wanting to escape the caregiving role*’ and ‘*acceptance of present circumstances and ability to plan ahead*’, were added to the Carer-QuALS framework in recognition of the varied coping strategies carers may adopt. The ‘*Control/choice*’ subtheme captures the ‘*uncertainty*’ and ‘*lack of control*’ often experienced by carers and includes ‘*making decisions to please others*’. The new ‘*Privacy*’ subtheme reflects carers’ need to have their own space and includes ‘*a sense of intrusion from others’*, such as healthcare professionals, visitors or friends.

### Psychological functioning: cognition

The theme ‘*Cognition*’ includes four subthemes: *Concentration*,* Understanding*,* Memory*, and *Thinking clearly & decision-making*. The act of ‘*seeking information to support understanding*’ was included within the ‘*Understanding*’ descriptor in recognition of the complex caregiving responsibilities in ALS. The concepts of ‘*cognitive burden*’, ‘*problem solving*’ and ‘*supporting decision-making*’ for the person with ALS was added to the descriptor of the subtheme ‘*Thinking clearly & decision-making*’. In addition, examples of caregiving tasks that require significant cognitive effort were added, including ‘*managing appointments’* and *‘complex equipment such as ventilatory aids and gastrostomy devices*’.

### Psychological functioning: feelings and emotions

The ‘*Feelings and Emotions*’ theme is the largest within the Carer-QuALS framework, comprising ten subthemes: *Happiness/sadness (depressed)*,* Psychological fatigue & energy*,* Hope/hopelessness*,* Grief*,* Anger/frustration*,* Worry*,* anxiety*,* stress*,* fear & calm*,* Safe/vulnerable*,* Wellbeing & life satisfaction*,* Guilt*, and *Loneliness & isolation*. The ‘*Grief*’ subtheme description was expanded from the QuALS framework to encompass the ‘*succession of losses*’ experienced when caring for someone with ALS. The ‘*Anger/frustration*’ subtheme was expanded to include feelings of ‘*resentment*’ towards the caregiving situation and context. The descriptor for ‘*Worry*,* anxiety*,* stress*,* fear and calm*’ was amended to reflect the hypervigilance and sense of urgency associated with providing 24/7 care. ‘*Contingency planning*’ and ‘*knowing who to call in a crisis*’ were added to the description of the ‘*Safe/vulnerable’* subtheme, in recognition of the sense of vulnerability that accompanies perceived total responsibility for caregiving in ALS.

### Psychological functioning: self-identity

The theme of ‘*Self-identity*’ comprises five subthemes: *Role identity*,* Identity as a person*,* Treated with dignity/respect*,* Self-concept*,* confidence & psychological self-worth*, and *Body image*. ‘*Role identity*’ captures the changes that occur when adopting the caregiving role, including a sense of duty or purpose. ‘*Identity as a person*’ includes seeing a person’s identity beyond caring. Closely related is the subtheme ‘*Treated with dignity/respect*’, whose descriptor was expanded to include ‘*feeling ignored’* or ‘*being treated as an extension*’ of the person with ALS. Advisory Group consultation supported retention of the ‘*Body image*’ subtheme, despite not being identified within review literature or PROMs. This subtheme was refined to reflect the ‘*change or loss in physical comfort with one’s body*’, ‘*self-consciousness*’, and alterations in body image resulting from ‘*deprioritising own needs*,* limited time for self-maintenance*,* and/or comfort eating*’.

### Social functioning: relationships

‘*Relationships*’ comprises six subthemes: *Social engagement*,* Support/support network*,* Impact on others*,* Friends & family*,* Belonging/connectedness*, and *Intimate relationships*. The ‘*Social engagement*’ descriptor was expanded to include ‘*social withdrawal*’ and ‘*reduced opportunities for participation due to the demands of the caring role*’. The ‘*Support/support network*’ subtheme was refined to include the ability to trust and confide in others, encompassing both practical and emotional support. The ‘*Friends and family*’ subtheme includes both the ability to form and maintain friendships and the broader effects of caregiving on relationship satisfaction. Finally, the ‘*Intimate relationships*’ subtheme underwent revisions to broaden the concept of ‘*emotional intimacy*’ to include ‘*empathy*,* connection and shared suffering*’ with the person with ALS, and the act of ‘*masking emotions*’ to protect others.

### Indexing PROMs and qualitative articles to the Carer-QuALS framework

**Fig. 4:**
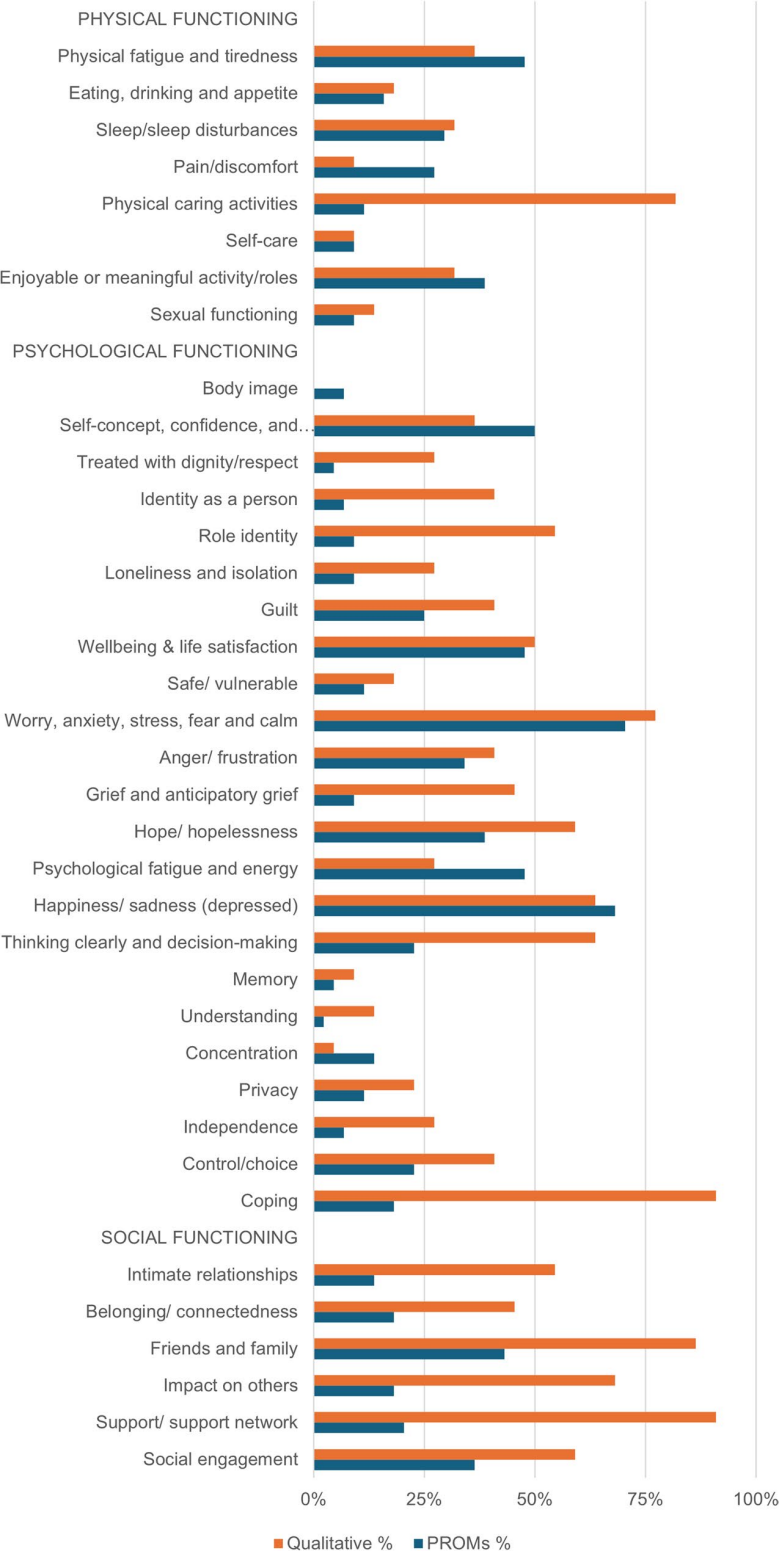
Frequency diagram illustrating coverage of all Carer-QuALS subthemes within HRQoL PROMs and qualitative articles included in this review. HRQoL = Health-Related Quality of Life. PROMs = Person-Reported Outcome Measures

The content of all PROMs and qualitative articles included within this review were indexed to the finalised Carer-QuALS framework (Additional File 6). Figure 4 provides a visual representation of the coverage of Carer-QuALS HRQoL subthemes across all PROMs and qualitative articles included in the review.

Overall, 21/44 (47.7%) PROMs covered at least one subtheme within all three broad HRQoL domains of physical, psychological and social functioning. Two domains were covered by 17/44 (38.6%) PROMs and 6/44 (13.6%) covered only one HRQoL domain within PROM items. Psychological functioning was the most commonly assessed HRQoL domain covered across all HRQoL PROMs within this review. The three most common subthemes covered by PROM content were all within this domain: ‘*Worry*,* anxiety*,* stress*,* fear & calm*’ 31/44 (70.5%) PROMs; ‘*Happiness/sadness*’ 30/44 (68.2%); and ‘*Self-concept*,* confidence & psychological self-worth*’ 22/44 (50%).

In contrast to PROMs, 19/22 (86.4%) of qualitative articles included in this review covered all three HRQoL domains (i.e., some elements of physical, psychological and social functioning). The remaining 3/22 (13.6%) of qualitative articles covered both psychological and social domains of functioning. The most common subthemes extracted from qualitative articles spanned all three HRQoL domains: ‘*Coping*’ 20/22 (90.9%), ‘*Support/support network*’ 20/22 (90.9%), ‘*Friends & family*’ 19/22 (86.4%) and ‘*Physical caring activities*’ 18/22 (81.8%).

The ZBI was the most frequently used PROM, appearing in 30/82 (68.2%) of articles included in this review. While the ZBI primarily addresses psychological and social aspects of HRQoL, studies that employed it used it in combination with up to six additional HRQoL PROMs to capture a broader picture of carers’ experiences. Coverage of differing HRQoL domains (i.e., physical, psychological and social functioning) was compared between carer and non-carer specific HRQoL PROMs identified within this review. Notably, social functioning was not represented in 2/9 (22%) of carer-specific, and 16/35 (45%) of non-carer specific HRQoL PROMs (Table 2).

**Table 2:** Coverage of HRQoL domains within non-carer and carer proms HRQoL = Health-Related quality of life. proms = Person-Reported outcome measures

Health-Related Quality of Life Domains	Non-Carer PROMs	Carer PROMs
Physical, Psychological and Social Functioning	14	7
Physical and Psychological Functioning	11	2
Psychological and Social Functioning	4	0
Physical and Social Functioning	0	0
Physical Functioning Only	1	0
Psychological Functioning Only	4	0
Social Functioning Only	1	0
TOTAL	35	9

## Discussion

This study presents the development of the Carer-QuALS, a comprehensive, evidence-based framework of HRQoL for informal carers of people with ALS. Carer-QuALS is based on evidence from quantitative and qualitative assessment of HRQoL of carers of people with ALS and ratified with an Advisory Group with lived experience. Carer-QuALS captures the complex and multidimensional impact of caring for someone with ALS on a carer’s own HRQoL. The breadth of subthemes identified within the framework highlights the extensive ways in which caring in ALS affects informal carers’ HRQoL. The framework can be used by researchers, clinicians, and patient advocacy groups for a variety of purposes including the selection of PROMs to measure HRQoL, guiding the development of future PROMs, and facilitating discussions between carers and clinicians. Further, all identified PROMs in the review were indexed to the Carer-QuALS framework. This can support the selection of the most appropriate PROMs based on their content coverage and is available as an additional resource for reference. A combination of ‘top-down’ and ‘bottom-up’ methods were utilised to develop Carer-QuALS. Traditional ‘top-down’ methods for developing a PROM or conceptual framework rely on research literature or expert input from clinicians or academics [36]. This study highlights the importance of incorporating lived experience perspectives in research (i.e., ‘bottom-up’ methods). Based on the feedback from carers a new subtheme ‘*Privacy*’ was added, in addition to further refinements to the content of the framework. The interactive and iterative development of Carer-QuALS through collaborative involvement highlights the potential limitations of relying solely on published literature (i.e., ‘top-down’ methods).

The indexing exercise undertaken within this review identified that no single PROM or qualitative article covered all HRQoL concepts identified within the Carer-QuALS framework. Such findings are suggestive that existing evidence and PROMs may be underestimating the true impact of caregiving on the HRQoL of carers in ALS. Within this study, the ZBI was the most frequently used PROM with ALS carers, potentially attributable to its extensive language validation [37]. Nevertheless, it does not assess physical functioning, which is a vital factor for ALS carers, given the commonly physically demanding nature of caregiving in ALS [5]. Further, social functioning was unrepresented in 22% of carer-specific and 45% of non-carer specific HRQoL PROMs within this review; a surprising finding given the impact of caregiving on carers ability to maintain a social life and relationships. The findings of this review suggest that there is a need for considerate choice of PROMs in future studies to ensure comprehensive coverage of important HRQoL concepts for carers in ALS.

This review identified a lack of HRQoL PROMs designed specifically for carers – both generally and specifically for ALS carers. Of all the HRQoL PROMs identified in this review, only nine (20.5%) were developed for use in general carer populations, and only one (2.3%), the Caregiver Network Scale (CNS) [35]was specifically developed for carers of people with ALS. The CNS covers all three broad domains of HRQoL, however, was used by only one study in this review and lacks comprehensive coverage of subthemes present within the Carer-QuALS framework. This review identifies the need to consider caregivers as a distinct population within future PROM research, both within the broader caregiving context and specifically in ALS, to ensure HRQoL assessment is relevant to their lived experience.

Whilst no formal assessment of study quality was included as part of this review, we noted some key limitations in the body of literature included in this review. A lack of transparency in methodological reporting was evident in many articles, particularly regarding the justification for PROM selection. In addition, information regarding the cognitive or behavioural status of individuals with ALS was frequently omitted. In some cases, individuals with cognitive and behavioural changes, and, by extension, their carers, were excluded from research entirely. This is a significant concern, as non-motor symptoms have been shown to contribute to carer strain over and above physical symptoms [11]. It is therefore plausible that the caregiving experience differs substantially in this context, highlighting the need for further research into the HRQoL of carers supporting individuals with ALS who present with cognitive and behavioural change.

Despite the methodological strengths of this review, it is not without its limitations. Firstly, although an extensive search strategy was utilised, it remains possible that relevant articles or PROMs listed in other databases were unidentified. Secondly, as previously acknowledged, the quality of included literature was not formally assessed as this is not deemed consistent with the aims of a scoping review [26]. Thirdly, whilst the Advisory Group were UK-based, the articles included in the review were not restricted by geographical location. Whilst there may be differences in the impact of informal caregiving on HRQoL as a consequence of factors including culture, country, health care resource allocation et cetera, the Carer-QuALS framework itself was international in scope. Finally, the Advisory Group consisted predominantly of female spousal carers. This may have influenced the revisions made to the Carer-QuALS framework. Future research could benefit from incorporating both international perspectives, including from caregivers who have differing relationships to the person with ALS for whom they provide care (i.e., parent, child, sibling, friend).

## Conclusion

The Carer-QuALS framework offers a comprehensive and condition-specific understanding of the HRQoL of informal carers of people with ALS. Developed through a robust, multi-stage process combining a scoping review and lived experience perspectives, it captures the extensive and multidimensional impact of caring across physical, psychological, and social functioning. By incorporating both ‘top-down’ and ‘bottom-up’ approaches, Carer-QuALS ensures a broad, internationally-informed, evidence-based foundation whilst being grounded in what matters to carers. Carer-QuALS addresses key gaps in existing tools and literature and can be used to support PROM selection, guide future PROM development, and facilitate meaningful dialogue between carers and clinicians. It provides a strong foundation for improving the visibility and support of informal carers within ALS care and research.

## Supplementary Information

Supplementary Material 1.

Supplementary Material 2.

Supplementary Material 3.

Supplementary Material 4.

Supplementary Material 5.

Supplementary Material 6.

Supplementary Material 7.

## Data Availability

The data supporting the conclusions of this article are included within the article and its additional file(s).

## Abbreviations

ALS: Amyotrophic Lateral Sclerosis
Carer-QuALS: Carer Quality of Life in Amyotrophic Lateral Sclerosis
HRQoL: Health Related Quality of Life
MND: Motor Neuron Disease
PRISMA: Preferred Reporting Items for Systematic Reviews and Meta-Analyses
PROMs: Person Reported Outcome Measures
QuALS: Quality of Life in Amyotrophic Lateral Sclerosis
QoL: Quality of Life
