# Supplementary material for: Understanding health-related quality of life of informal carers in amyotrophic lateral sclerosis: a scoping review and conceptual framework
Source: Health Qual Life Outcomes. 2025 Sep 29;23:90. doi: 10.1186/s12955-025-02427-2 (PMC12482542; doi:10.1186/s12955-025-02427-2)
Supplement: Supplementary file 1 — Supplementary Material 1. [file 12955_2025_2427_MOESM1_ESM.docx]

**Additional File 1: Search Strategy & Hierarchical Screening Tool**

MEDLINE via Ovid

| **HR-QoL** |  | **Carers** |  | **MND** | **Yield** |
| --- | --- | --- | --- | --- | --- |
| (HRQL or HRQoL or QL or QoL).mp. or quality of life.mp. or (health index* or health indices or health profile*).mp. or health status.mp. | AND | exp Caregivers/ OR carer*.mp. OR caring.mp. | AND | (ALS or "Amyotrophic lateral sclerosis").mp. or exp Motor Neuron Disease/ or Motor neuron* disease*.mp. or Motor neuron disease*.mp. or MND.mp. |  |
| **629,844** |  | **111,309** |  | **99,484** | **291** |

EMBASE via Ovid

| **HR-QoL** |  | **Carers** |  | **MND** | **Yield** |
| --- | --- | --- | --- | --- | --- |
| (HRQL or HRQoL or QL or QoL).mp. or quality of life.mp. or (health index* or health indices or health profile*).mp. or health status.mp. | AND | (carer* or caring or carergiv* or care-giv* or informal care* or family care* or spouse care*).mp. | AND | (ALS or "Amyotrophic lateral sclerosis").mp. or exp Motor Neuron Disease/ or Motor neuron* disease*.mp. or Motor neuron disease*.mp. or MND.mp. |  |
| **1,005,427** |  | **126,343** |  | **115,867** | **297** |

CINAHL via EBSCO

| **HR-QoL** |  | **Carers** |  | **MND** | **Yield** |
| --- | --- | --- | --- | --- | --- |
| ("HRQL" or "HRQoL" or "QL" or "QoL" or “quality of life” or “health index*” or “health indices” or “health profile*” or “health status”) | AND | (MH "Caregivers") OR carer* OR caring OR carergiv* OR care-giv* | AND | (MH "Motor Neuron Diseases+") OR ALS OR “Amyotrophic lateral sclerosis” OR Motor neuron* disease* OR Motor neuron disease* |  |
| **327,212** |  | **104,744** |  | **17,634** | **127** |

| **Host** | **Database** | **Dates Covered** | **Date Searched** | **Search Yield** |
| --- | --- | --- | --- | --- |
| Ovid | MEDLINE(R) and Epub Ahead of Print, In-Process & Other Non-Indexed Citations, Daily and Versions(R) | 1946 - Date | 08/03/2024 | 291 |
| Ovid | Embase | 1974 - Date | 08/03/2024 | 297 |
| EBSCO | CINAHL | 1974 - Date | 08/03/2024 | 127 |
| **TOTAL YIELD** | | | | **715** |

Hierarchical Screening Tool


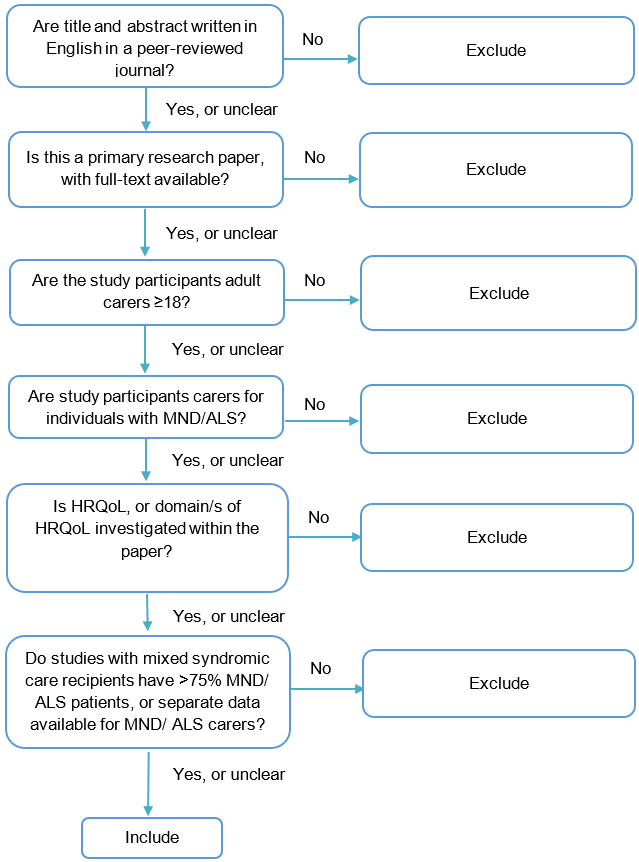


| **Additional File 1: Hierarchical Screening Tool (Polanin et al., 2019)**  Hierarchical screening tool developed according to predetermined eligibility criteria by one researcher to support systematic and reproducible screening strategy. Domains of HRQoL refer to physical, psychological and social functioning.  ALS = Amyotrophic Lateral Sclerosis, HRQoL = Health-Related Quality of Life, MND = Motor Neuron Disease, PROM = Patient Reported Outcome Measure. |
| --- |
