# Supplementary material for: Understanding health-related quality of life of informal carers in amyotrophic lateral sclerosis: a scoping review and conceptual framework
Source: Health Qual Life Outcomes. 2025 Sep 29;23:90. doi: 10.1186/s12955-025-02427-2 (PMC12482542; doi:10.1186/s12955-025-02427-2)
Supplement: Supplementary file 2 — Supplementary Material 2. [file 12955_2025_2427_MOESM2_ESM.docx]

**Additional File 2: Advisory Group Consultation**

**Recruitment**

The opportunity to participate in ratifying the Carer-QuALS framework was advertised from November 2024 to January 2025 within clinical networks (Sheffield Neurological Outreach Therapy Service, Sheffield MND Care and Research Centre, and Oxford MND Care and Research Centre) and via the Motor Neuron Disease Association (MNDA) Research Development Team. A recruitment poster was circulated to the above networks via email and carers were offered £50 reimbursement for two hours of their time (30-minutes for preparatory work, 90-minutes for the online session). Advisory Group members were invited to participate if they were current or past informal (unpaid) carers of someone with ALS. Carers expressed interest to participate via email and were contacted by the primary researcher to confirm interest and discuss additional needs, support required to participate in the online session, and preferred timings. Carers were given the opportunity to speak to the primary researcher via telephone if they would prefer and to promote inclusivity. Nine informal carers were recruited from across seven UK counties. The carer cohort comprised eight current and one bereaved carer. All carers were spousal carers (88.9% female).

**Preparatory Materials**

Two weeks before the sessions, a preparatory email with three short videos were sent via email to Advisory Group members. The videos covered the following topics: (1) The Better Outcomes in Motor Neuron Disease (BOND) Team, (2) Health-Related Quality of Life, and (3) What to expect from the sessions. Carers were again given the opportunity to speak to primary researcher via telephone to discuss any queries or concerns.

**Online Sessions**

Three different 90-minute online sessions were facilitated on the 26^th^, 27^th^ and 28^th^ February 2025. Sessions were facilitated in the day and in the evening to accommodate carers differing availability (some carers had full-time work commitments and were only available in the evening, one carer felt able to participate 1:1 but not in a group session). Online sessions were led by a highly experienced researcher specialising in quality-of-life research and lived experience consultation (JC) and an occupational therapist (RB). Sessions began with an introduction to the BOND team and Advisory Group members, and housekeeping. Within housekeeping the following principles were shared with carers: (1) everything shared within sessions will stay within the group and future outcomes anonymised, (2) everyone is given the opportunity to share, (3) there are no wrong answers, all views and opinions are valid, (4) carers can take a break whenever you need and/or turn their cameras off should they need to, and (5) the session will be recorded for note-taking purposes. The Carer-QuALS project, key terminology and the structure of the hierarchical framework was outlined. Session one, facilitated 13:00-14:30 on 26^th^ February was attended by four carers and began with the physical functioning theme. Session two, facilitated 18:00-19:30 on 27^th^ February was attended by four carers and was conducted in reverse order of themes, beginning with the psychological functioning theme. Session three, facilitated 09:30-11:00 on 28^th^ February was attended by one carer and began with the social functioning theme.

**Dissemination & Communication**

Carers were sent a thank you email acknowledging the value of their participation to the project. Once Advisory Group amendments were integrated into the Carer-QuALS framework, this was circulated to carers via email. This email included a high-level summary of changes made to the framework, a non-compulsory invitation to provide feedback within a two-week period about whether Advisory Group members felt their insights were sufficiently captured. While all feedback from sessions was anonymised, the contributions of carers from all three sessions will be acknowledged under the group name ‘*the Carer-QuALS Advisory Group*’.
