# Supplementary material for: Understanding health-related quality of life of informal carers in amyotrophic lateral sclerosis: a scoping review and conceptual framework
Source: Health Qual Life Outcomes. 2025 Sep 29;23:90. doi: 10.1186/s12955-025-02427-2 (PMC12482542; doi:10.1186/s12955-025-02427-2)
Supplement: Supplementary file 3 — Supplementary Material 3. [file 12955_2025_2427_MOESM3_ESM.docx]

**Additional File 4: PROMs Identified in Stage 1 Searches**

| **#** | **PROM Name** | **PROM Abb.** | **Reviewed (Y/N)** | **Exclusion Rationale** |
| --- | --- | --- | --- | --- |
| 1 | Acceptance of Illness Scale | AIS | Y | 7 |
| 2 | Affiliate Stigma Scale | ASS | Y | 6 |
| 3 | ALS Cognitive Behavioural Screen | ALS CBS | Y | 4 |
| 4 | ALS Depression Inventory-12 Item | ADI-12 | Y | 7 |
| 5 | Amnestic Comparative Self-Assessment | ACSA | Y | 0 |
| 6 | Beck Depression Inventory | BDI | Y | 7 |
| 7 | Beck Depression Inventory-II | BDI-II | Y | 7 |
| 8 | Beck Hopelessness Scale | BHS | Y | 7 |
| 9 | Big Five Questionnaire | BFQ | N | N/A |
| 10 | Brief Symptom Inventory | BSI | Y | 7 |
| 11 | Burden Scale for Family Caregivers | BSFC | Y | 7 |
| 12 | Caregiver Burden Inventory | CBI | Y | 7 |
| 13 | Caregiver Burden Scale | CBS | Y | 7 |
| 14 | Caregiver Network Scale | CNS | Y | 7 |
| 15 | Caregiver Reaction Assessment | CRA | N | N/A |
| 16 | Caregiver Strain Index | CSI | Y | 7 |
| 17 | Caregiver Strain Scale | CSS | N | N/A |
| 18 | Carer Quality of Life | CarerQoL | Y | 7 |
| 19 | Chalder Fatigue Scale | CFS | Y | 7 |
| 20 | Chalder Fatigue Scale - Physical Fatigue Subscale | CFS - Physical | Y | 7 |
| 21 | Close Persons Questionnaire | CPQ | Y | 7 |
| 22 | Coping Inventory for Stressful Situations | CISS | N | N/A |
| 23 | Cost of Care Index | CCI | Y | 7 |
| 24 | Depression, Anxiety Stress Scale | DASS-21 | Y | 7 |
| 25 | Dyadic Adjustment Scale - Dyadic Subscale | DAS | Y | 7 |
| 26 | EuroQoL Visual Analogue Scale | EQ VAS | Y | 0 |
| 27 | EuroQoL-5 Dimensions | EQ-5D-5L | Y | 7 |
| 28 | Existential Well-Being Subscale from the McGill Quality of Life Questionnaire | EWBS | Y | 7 |
| 29 | Family Cohesions and Adaptability III | FACES III | Y | 5 |
| 30 | Folkham's 4-item Measure of Finding Positive Meaning in Caregiving | N/A | N | N/A |
| 31 | Folkham's Measure of Caregiver Satisfaction | N/A | N | N/A |
| 32 | General Health Questionnaire-12 | GHQ-12 | Y | 7 |
| 33 | Hospital Anxiety & Depression Scale | HADS | Y | 7 |
| 34 | Hamilton Anxiety Scale | HAMA | Y | 4 |
| 35 | Hamilton Depression Scale-17 | HAMD-17 | Y | 4 |
| 36 | Idler Index of Religiosity | IIR | Y | 5 |
| 37 | Job Content Questionnaire | JCQ | Y | 5 |
| 38 | Life Satisfaction Checklist | LiSat-11 | Y | 7 |
| 39 | Langer Mindfulness Scale | LMS | Y | 5 |
| 40 | Manne Scales of Positive and Negative Dyad Support | N/A | N | N/A |
| 41 | Marital Intimacy Scale | MIS | Y | 5 |
| 42 | McGill Quality of Life Questionnaire | MQOL | Y | 7 |
| 43 | McGill Quality of Life Questionnaire Single-Item Scale | MQOL-SIS | Y | 0 |
| 44 | Multidimensional Scale of Perceived Social Support | MPSS | Y | 7 |
| 45 | Patient Health Questionnaire-9 | PHQ-9 | Y | 7 |
| 46 | Profile of Mood States - Short Form | POMS-SF | Y | 7 |
| 47 | Purpose in Life Test | PIL | Y | 7 |
| 48 | Quality of Care | QoC | N | N/A |
| 49 | QoL Enjoyment & Satisfaction Questionnaire Short Form | Q-LES-Q-SF | Y | 7 |
| 50 | Quality of Life in Life-Threatening Illness Family Carer Version | QOLLTI-F | Y | 7 |
| 51 | Quality of Life Inventory | QoLI | N | N/A |
| 52 | Relationship Satisfaction Scale | N/A | N | N/A |
| 53 | Revised Scale for Caregiving Self-Efficacy - Controlling Upsetting Thoughts Subscale | RSCSE-Contr | Y | 5 |
| 54 | Revised Scale for Caregiving Self-Efficacy | RSCSE-Resp | Y | 5 |
| 55 | Satisfaction With Life Scale | SWLS | Y | 7 |
| 56 | Schedule for the Evaluation of Individual Quality of Life–Direct Weight | SEIQoL-DW | Y | 3 |
| 57 | Self-Rating Anxiety Scale | SAS | Y | 7 |
| 58 | Self-Rating Depression Scale | SDS | Y | 7 |
| 59 | Short Form-12 | SF-12 | Y | 7 |
| 60 | Short Form-36 | SF-36 | Y | 7 |
| 61 | Short Form-36 Version 2 | SF-36 V2 | Y | 7 |
| 62 | Short Form-36 Mental Component Summary | SF-36 MCS | Y | 7 |
| 63 | Short Form-8 | SF-8 | Y | 7 |
| 64 | Social Problem-Solving Inventory Revised | SPSI-R | N | N/A |
| 65 | Social Support Questionnaire | SSQ | Y | 5 |
| 66 | State-Trait Anxiety Inventory-Y | STAI-Y1 & Y2 | Y | 7 |
| 67 | State-Trait Anxiety Inventory-Y1 | STAI-Y1 | Y | 7 |
| 68 | State-Trait Anxiety Inventory-X | STAI-X | Y | 7 |
| 69 | Systems of Belief Inventory | SBI-15R | Y | 5 |
| 70 | The Level of Care Index | N/A | N | N/A |
| 71 | The Life Rating Scale | N/A | N | N/A |
| 72 | The Spiritual Perspective Scale | SPS | Y | 5 |
| 73 | Visual Analogue Scale for Carer Burden | N/A | N | N/A |
| 74 | Ways of Coping Questionnaire | WOCQ | Y | 7 |
| 75 | World health organisation quality of life-BREF | WHOQOL-BREF | Y | 7 |
| 76 | Zarit Burden Interview | ZBI | Y | 7 |

| **Additional File 4: PROMs Identified in Stage 1 Searches**  All PROMs extracted from quantitative or mixed-methods articles with full names and abbreviations. Reason for PROM exclusion: (0) Not a multi-item PROM, (1) Not available in English, (2) Not freely available, (3) Not a self-report measure and cannot be used systematically across a population, (4) Does not yield quantitative score/s, (5) 1≤ item covers an aspect of HRQoL, (6) Not a validated version or adaptation of an existing PROM, (7) Include PROM.  Abb. = Abbreviation, N = No, PROM = Person Reported Outcome Measure, Y = Yes. |
| --- |
