# Supplementary material for: Understanding health-related quality of life of informal carers in amyotrophic lateral sclerosis: a scoping review and conceptual framework
Source: Health Qual Life Outcomes. 2025 Sep 29;23:90. doi: 10.1186/s12955-025-02427-2 (PMC12482542; doi:10.1186/s12955-025-02427-2)
Supplement: Supplementary file 7 — Supplementary Material 7. [file 12955_2025_2427_MOESM7_ESM.docx]

**Additional File 5: Advisory Group Amendments**

|  | **Subtheme** | **Feedback** | **Ratification** |
| --- | --- | --- | --- |
| **Activity** | Physical Caring Activities | Different physical activities included in caring role: toileting, shopping, managing medications, cooking | Add toileting |
|  |  | Pressure relief 'pressure lifting', repositioning and wheelchair maintenance a proposed addition to physical caring activities | Add repositioning and pushing/ maintaining wheelchair |
|  |  | Other physical tasks are adopted due to the physical decline of the person who is cared for, e.g. DIY, gardening | No changes - these are not physical caring activities |
|  | Self-Care | Agreement with self-neglect addition | Agreement with self-neglect addition |
|  | Enjoyable or Meaningful Activity | Change in ability to engage in previously meaningful activities (e.g. no longer able to cycle do to risk of injury and dependence of cared for person) | CHANGE' in ability to do activities... rather than just 'ability to...' |
|  |  | Lack of time for meaningful activity given 24/7 nature of caring role +/- working role | This is encompassed in hypervigilance - no change |
|  |  | Finding time and 'space' for meaningful activity/ respite. Life is on hold | No change, this is not a phrase that would be internationally recognised/ familiar |
|  |  | Ability to engage in work | Add 'perceived impact on work colleagues and impact of caring on work relationships' to 'impact on others' in social functioning theme |
|  |  | Ability to engage in health-promoting behaviours (e.g. going to the gym/ exercise) | Add 'health-promoting' behaviours to descriptor to emphasise the sacrifices made by carers |
|  | Communication | Caring for someone that you have a deep relationship with, the communication changes. Loss of voice, use of technology. Impact on general conversation. Mis-reading facial expressions. Accommodating and adjusting communication | Omit communication subtheme. Carers are describing the emotional impacts of their care recipients' impaired communication. This is not about the carers' own physical ability to communicate |
|  |  | Pressure to understand person with MND | As above |
|  |  | Rapid adjustment to change in communication | As above |
|  | Upper Mobility | Agreement with omitting this for carers | Omit upper mobility |
|  | Lower Mobility | Agreement with omitting this for carers | Omit lower mobility |
| **Physical Health** | Eating, Drinking, Swallowing and Appetite |  | Removal of 'swallowing' from subtheme title |
|  |  | Impact on ability to eat socially e.g. PEG, length of time taken for care recipient to eat | Add ability to eat with others/ eat socially |
|  |  | Guilt about ability to eat when this declines in care recipient. Other emotional aspects of eating behaviours, e.g. comfort eating, eating in secret without care recipient | Add emotional aspects of eating - e.g. comfort eating, guilt, secret eating |
|  |  | Fear of care recipient choking | No change - fear is encompassed with hypervigilance |
|  |  | Food becoming functional, rushed, not enjoyable | No change |
|  |  | Having the time and energy to cook | No change - encompassed in mental and physical fatigue |
|  | Cough | Impact of coughing on the carer | No change - omit cough |
|  | Secretions | Agreement with omitting this for carers | Omit secretions |
|  | Breathing | Agreement with omitting this for carers | Omit breathing |
|  | Speech | Agreement with omitting this for carers | Omit speech |
| **Relationships** | Social Engagement | Social withdrawal and forced social isolation | CHANGE of social network - the descriptor for 'social engagement' and 'support/social network' needs to be altered. E.g. 'interest in others' does not feel appropriate for carers - need to emphasise the change in their social network due to reduced opportunity to socialise |
|  |  | Prioritising social needs of care recipient, guilt surrounding leaving the home or socialising | No change |
|  |  | Social media as social engagement - social engagement changing | No change |
|  | Impact on Others | Ability to continue to work, loss of engagement with work colleagues | Add 'perceived impact on work colleagues and impact of caring on work relationships' |
|  | Friends and Family | Burden of visitors e.g. washing, making beds, additional cooking | Hosting/ entertaining family and friends' added to 'family and friends' subtheme |
|  |  | Loss of friendships and connection | No change, 'losing friends' is already in descriptor |
|  | Intimate Relationships | Loss of emotional intimacy, focus on 'doing' functional caring tasks | ‘Change or loss’ of emotional intimacy |
|  |  | Constant masking of emotions - agree with masking | No change to masking |
| **Autonomy** | Privacy (New subtheme) | Taking myself away/upstairs, having a little bit of time. Having time for yourself or feeling that you don't have time or a safe space anymore. Being able to have own space - individually, mentally, emotionally | Agreement with addition of 'privacy' subtheme and descriptors |
|  |  | Revolving door, constant visitors - intrusive. Not being in control when people visit, arrive, leave. Not being able to shut the door - open door environment (without choice). Home feels like a hospice | Ensure intrusiveness is sufficiently encompassed in 'privacy' descriptor |
|  |  | Loss of 1:1 time with care recipient due to intrusive/ invasive presence of constant visitors (HCPs and friends) | No change |
|  |  | Separateness sought to deal with intrusiveness/ exhaustion. Privacy from the care recipient also craved due to 24/7 nature of caregiving role/ disproportionate dependency on primary informal caregiver. Space/ time is needed away from the person you care for | No change - already encompasses in 'ability to take a break and seek respite from caring responsibilities' |
|  | Control/choice | When the care recipient makes a choice that is conflicting with the choices the carer would make. This disempowers the carers choices and sense of control. Dealing with disconnect. This could also encompass differences of opinions with HCPs | Amend brackets after 'Uncertainty and lack of control' to encompass lack of control of choices of care recipient |
|  |  | Control over choices to manage one's life' and 'uncertainty..' could sit together in one descriptor | No change |
|  | Coping | Agreement with addition of the word 'adapting to' | Agreement with addition of 'adapting to' in descriptor |
|  |  | Agreement with 'escape' and avoidance descriptor | Agreement with addition of 'escape and avoidance' descriptor |
|  |  | For coping strategies, the opposite of 'being present' can also be true i.e. getting out of the house or doing a hobby that creates a space/ break/ escape from caregiving temporarily | Expand coping strategies descriptor to encompass this |
|  |  | Seeking information and knowledge, advocacy and having a voice in the MND community as a coping strategy. Trying to make a difference or make things better for others | List advocacy and seeking information under coping strategy |
|  |  | Managing expectations and accepting 'new normal' as a coping strategy | No change |
|  |  | Setting parameters/ boundaries around information provision and exposure to future planning. For others future planning and anticipation of change itself was coping strategy, creating sense of control and preparedness. Knowing when and when not to look ahead. Balance between present and future focus | Add 'This may include acceptance of present circumstances and ability to plan ahead' to coping subtheme. |
| **Feelings and Emotions** | Pride/ embarrassment/ guilt/ shame | (Email feedback from a carer) As a carer, I feel guilt for wanting time away, guilt for having those feelings of wanting to 'escape', guilt for wanting to go to work, and guilt for feeling angry and frustrated with them or their condition, which they didn't ask for either and isn't their fault | Emphasise guilt with use of examples in this subtheme descriptor |
|  |  | Embarrassment/ pride/ shame - not a prominent emotion described within carer narratives | Rename this theme 'guilt' - embarrassment, pride and shame do not appear relevant to carer narrative |
|  | Anger/frustration | Resentment does not necessarily apply to each of the following words, better encompassed in first descriptor - and then add that this can be towards ALS, the caregiving situation, the context. | Move resentment into first descriptor |
|  |  | Add frustration towards health services as an example context | Add health services as an example context for this descriptor |
|  | Wellbeing & life satisfaction | Caregiving not perceived to be fulfilling. It is not a choice - feelings of entrapment in the caregiving role and knowing the outcome of inevitable decline of the care recipient. Can still feel like a team with the care recipient but it’s not fulfilment | Remove the word fulfilment |
|  |  | Can identify with a sense of satisfaction when performing care tasks that support or help the care recipient. And the sense of wanting to provide the best care possible | No change |
|  |  | Reciprocity not the right term. Caregiving feels one sided. Not everyone is a spousal carer that would imply a more balanced foundation for reciprocity. Other caregiving relationships may have less inherent balance/ reciprocity at baseline and therefore does not feel like an appropriate term | Remove reciprocity. |
|  | Worry, anxiety, stress, fear and calm | The current descriptors do not encompass a constant sense of pressure, time pressure - too much to do and too little time. Urgency associated with swift disease progression. Pressure from the care recipient and from family members also is not captured. This could be called 'emotional pressure'. Pressure also extends to pressure placed on oneself to get things right for the care recipient (e.g. anticipating their needs, getting the right support, at the right time, pressure of being primary informal caregiver) | Add pressure to subtheme descriptor |
|  | Loneliness and Isolation | Add 'avoided' to the description | No change |
|  |  | Physical isolation, does not encompass sense of mental/ emotional isolation | No change |
|  | Safe/vulnerable | Importance of contingency planning to create a sense of psychological safety for carers | Add contingency planning to descriptor |
|  |  | Family and friends could be included in the descriptor about relying on others | Add friends and family to descriptor about relying on others |
|  | Anticipatory Grief | Loss in MND is more than anticipatory grief, caring in MND encompasses a succession of losses, e.g. loss of communication | Add grief to subtheme title 'Grief and Anticipatory Grief' |
|  |  | As above | Add succession of losses to grief descriptor |
| **Cognition** | Understanding | Agreement with adding 'seeking information' in the descriptor | Agreement with addition of 'seeking information' in descriptor |
|  |  | Learning to use complex new equipment | Add learning to understanding descriptor |
|  | Memory | Remembering how to use equipment (e.g. NIV mask, wheelchair adjustments, manual handling aids, PEG) more important than medications in MND. Complexity of aids, equipment and daily care routine in MND. Remembering appointments also important. Using routines to support memory, due to the complexity of care tasks in MND | Add equipment, appointments, care routine to descriptor |
|  | Thinking clearly and decision-making | Agreement with adding 'problem solving' and 'supporting decision making of care recipient' to descriptor | Agreement with proposed addition to descriptor |
|  |  | Can insight and self-awareness of the likely impact of stress of caring on thinking skills be added to descriptors? Maybe alongside cognitive burden. E.g. 'the ability to recognise impact of caring on cognitive function, this may include a sense of cognitive burden' | No change - insight/ metacognition is not a component of HRQoL |
|  |  | Organising appointments, multi-agency coordination, organising appointments (PA) | Encompass cognitive caring tasks within cognitive burden descriptor |
| **Self-Identity** | Treated with dignity/respect | Agreement with addition of 'being treated as extension of care recipient/ being ignored' | Agreement with proposed addition to descriptor |
|  |  | Being taken for granted' - having to accommodate for care recipient, sense of duty that others fail to appreciate. Having to put things on hold. Assumption of constant availability for care recipient by others (e.g. HCPs) | No change - already encompasses in 'being treated with dignity and respect' |
|  | Self-concept, confidence, and psychological self-worth | Disagreement with the descriptor encompassing personal growth: Sense of shrinking rather than growing. Suppressing parts of your life or parts of yourself to accommodate the caring role. Supporting character in someone else's main story. Disagreement with empowerment - caring is a necessity without choice | Remove growth from descriptor. |
|  |  | Does 'knowing oneself as identity changes' fit better in the 'role identity' subtheme | No change |
|  |  | Lack of confidence is not currently captured, self-doubt, exacerbated by encounters with HCPs. Lack of confidence in other areas of life outside of caring role (e.g. parenting, work) | Add 'change in sense of self-confidence' |
|  | Body Image | Keep: impact of deprioritising own health needs, comfort eating, lack of time to exercise. Loss of sense of comfort or confidence in own physical body. Time for self-maintenance. Impact of perception of own body | Keep body image! Expand descriptor with some of the carer narrative on the left. |
|  |  | Embarrassment and shame strongly expressed when discussing poor body image | No change - these are feelings and emotions. |
